# Rational Targeting and gRNA Design for Enhancing Quorum Quenching in *Pseudomonas aeruginosa* PAO1

**DOI:** 10.34133/csbj.0089

**Published:** 2026-05-04

**Authors:** Javier Alejandro Delgado-Nungaray, Luis Joel Figueroa-Yáñez, Eire Reynaga-Delgado, Mario Alberto García-Ramírez, Orfil Gonzalez-Reynoso

**Affiliations:** ^1^ Chemical Engineering Department, University Center for Exact and Engineering Sciences, University of Guadalajara, Guadalajara 44430, Jalisco, Mexico.; ^2^ Industrial Biotechnology Unit, Center for Research and Assistance in Technology and Design of the State of Jalisco, A.C. (CIATEJ), Zapopan, Jalisco, Mexico.; ^3^ Pharmacobiology Department, University Center for Exact and Engineering Sciences, University of Guadalajara, Guadalajara 44430, Jalisco, Mexico.; ^4^ Electronics Department, University Center for Exact and Engineering Sciences, University of Guadalajara, Guadalajara 44430, Mexico.

## Abstract

Quorum quenching enzymes (QQEs) are a promising antivirulence strategy by disrupting quorum sensing (QS), a mechanism that regulates biofilm formation in *Pseudomonas aeruginosa*, a key factor in adaptive antibiotic resistance. In this study, a systems biology approach based on the genome-scale metabolic model iJD1249 and flux balance analysis simulating growth in Luria–Bertani medium and QS-activating conditions was used to identify gene targets associated with enhanced endogenous PvdQ production, the most representative QQE. Following gene–protein–reaction filtering of nonessential genes involved in QS-related pathways, a rational CRISPR-Cas9 guide RNA (gRNA) design strategy was implemented to support future genome editing validation. gRNAs were first generated using CHOPCHOP, considering on-target efficiency, mismatch number, and self-complementarity. A semiquantitative scoring system based on gRNA efficiency parameters was applied to prioritize top gRNAs, followed by secondary structure prediction using RNAfold. Simulations identified 10 genes associated with PvdQ maximization. Among them, *fabI*, involved in palmitate biosynthesis II, emerged as the most promising target. Its knockout is predicted to limit acyl-acyl carrier protein intermediate availability required for QS signal biosynthesis, potentially influencing *pvdQ* expression through metabolic redistribution. To avoid unintended pyoverdine enhancement, which is directly influenced by PvdQ, gRNAs were also designed to target *pvdH*. From an initial set of 78 and 146 sequences for *fabI* and *pvdH*, respectively, gRNA No. 12 (*fabI*) and gRNA No. 16 (*pvdH*) were identified as the most efficient gRNA hits for gene knockout. Experimental validation is required to confirm the predicted metabolic effects and provide deeper insights for QQ-based strategies against *P. aeruginosa*.

## Highlights

• Systems biology approach identified 10 gene targets that enhance endogenous PvdQ
• *fabI* is the most promising knockout target for increasing PvdQ and promoting QQ
• Targeting *pvdh* for knockout prevents pyoverdine increase from PvdQ overproduction
• gRNAs designed based on Cas9 efficiency, scoring system, and RNA secondary structure

## Graphical Abstract

**Figure:**
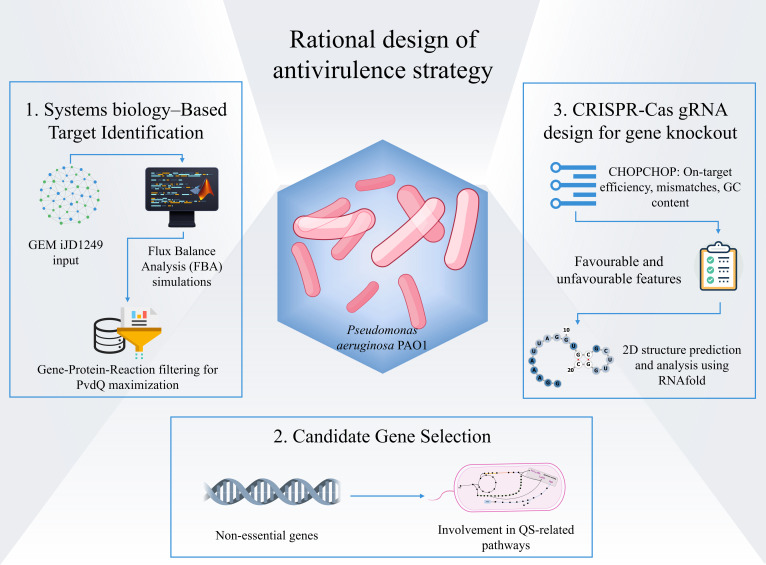

## Introduction

Antimicrobial resistance (AMR) is a major global health threat that compromises our ability to treat microbial infections, projected to cause 10 million deaths annually by 2050, and to manage the economic impact of drug resistance [1]. In light of the AMR threat, *Pseudomonas aeruginosa* was classified as a high-priority pathogen in the updated bacterial priority pathogens list by the World Health Organization [2] due to its multidrug resistance (MDR) and its substantial impact on human health, which is exacerbated by limited treatment options. *P. aeruginosa* increases the mortality rate by 30% in patients with nosocomial infections, including pneumonia, catheter-related infections, surgical site infections, implant-associated infections, intensive care unit infections, and immunocompromised patients [3,4].

The adaptive antibiotic resistance development by *P. aeruginosa* depends, in part, on the biofilm matrix, a bacterial aggregate encapsulated within self-synthesized extracellular polymeric substances (EPS), resulting in strains 10 to 1,000 times more antibiotic-resistant than planktonic cells [5]. Biofilm reduces antibiotic penetration by acting as a diffusion barrier that promotes bacterial growth heterogeneity. Oxygen and nutrient gradients contribute to the formation of persister cells, reducing the effectiveness of growth rate-dependent antibiotics. The presence of EPS and extracellular DNA (eDNA) in the biofilm microenvironment enables bacterial phase variation between resistant and nonresistant phenotypes and facilitates the horizontal transfer of biofilm-specific genes [6].

Biofilm development and structural integrity rely on quorum sensing (QS), a cell density-dependent communication system, which entails the production, secretion, accumulation, and recognition of signaling molecules known as autoinducers (AIs) [7]. QS in *P. aeruginosa* is composed of 4 hierarchical systems: Las, Rhl, Pqs, and Iqs. Each system consists of a transcriptional activator (LasR, RhlR, PqsR, and IqsR) and its synthase (LasI, RhlI, PqsABCD, and ambABCDE or PchABCDEF, respectively). However, the inclusion of Iqs as the fourth system is still debated, as does the identification of its specific synthase. The Las and Rhl systems are both based on N-acyl-homoserine lactones (AHLs). The Las system is considered to be at the top of the QS hierarchy and relies on N-3-oxo-dodecanoyl-homoserine lactone (3O-C_12_-HSL), while the Rhl system relies on butyryl-L-homoserine lactone (C_4_-HSL). The Pqs system is based on 2-heptyl-3-hydroxy-4(1H)-quinolone (also known as *Pseudomonas* quinolone signal [PQS]), and the Iqs system is associated with the 2-(2-hydroxyphenyl)-thiazole-4-carbaldehyde (also known as integrated quorum sensing signal [IQS]) [8].

In light of the established importance of QS in biofilm formation and the pathogenesis of *P. aeruginosa*, disrupting QS has emerged as a promising antimicrobial strategy known as quorum quenching (QQ), which can be achieved by degrading AIs through QQ enzymes (QQEs), down-regulating the expression of AI synthases, or blocking AI receptors. This approach, in contrast to the use of antibiotics, targets virulence with reduced evolutionary pressure, thereby potentially mitigating AMR development [9]. *P. aeruginosa* is capable of cleaving its AHLs through the QQ acylases PvdQ, QuiP, and HacB that catalyze the irreversible hydrolysis of AHLs at the amide bond between the L-homoserine lactone and the acyl side chain, releasing fatty acid and homoserine lactone. The endogenous activity of these QQEs is thought to regulate AI production, enabling bacteria to adjust QS and adapt to changing environmental conditions [10–13].

The race to develop antivirulence drugs has demonstrated the therapeutic efficacy of PvdQ, the most representative QQE in *P. aeruginosa* PAO1, in a mouse pulmonary infection model [14]. Recent research has also focused on enhancing its biochemical and kinetic enzymatic properties [15]. However, PvdQ also plays a well-known role in the maturation of siderophore pyoverdine, a virulence factor in *P. aeruginosa*. Consequently, some studies have explored PvdQ inhibition as a strategy to limit pyoverdine production [16,17]. Thus, the optimal strategy would entail PvdQ overproduction, while considering that its expression could reduce one virulence factor but unintentionally enhance another, raising important biosafety concerns. To address this, a loss-of-function research strategy can be adopted to mitigate siderophore production, in which the *pvdH* gene (PA2413) is selected for knockout, as mutants lacking this gene are incapable of producing pyoverdine [18].

Computational modeling of biological systems is key to identifying new potential targets to combat MDR. Since PvdQ production is integrated within a complex metabolic network, a systems biology approach enables an in-depth understanding of bacterial metabolism, guiding effective genetic modifications to enhance metabolite production [19,20]. In our previous work [21], we developed the genome-scale metabolic model (GEM) iJD1249 that includes all available metabolic information about *P. aeruginosa* PAO1, which incorporates mass-balanced biochemical reactions and gene–protein–reaction (GPR) associations, including pathways related to QS and QQEs PvdQ and QuiP.

Following the identification of new DNA targets, genetic engineering techniques such as Clustered Regularly Interspaced Short Palindromic Repeats (CRISPR) and CRISPR-associated (Cas) proteins have been used to target QS genes related to decreasing biofilms [22,23]. Effective application of this strategy requires the optimal design of guide RNAs (gRNAs) that account for on-target efficiency, off-target effects, sequence mismatches, and RNA secondary (2D) structure to maximize Cas endonuclease activity. In genome engineering, the CRISPR-Cas9 system commonly comprises a single guide RNA (sgRNA) composed of a 20-nucleotide (nt) gRNA, which specifies the target DNA site, fused to a scaffold region that interacts with the Cas9 protein, followed by a transcription termination residue (TTTT). The complete structure of the sgRNA is organized as 5′-20 nt gRNA–scaffold–TTTT-3′ [24,25]. Importantly, *P. aeruginosa* PAO1 has been found to carry an orphan CRISPR system that should not interfere with exogenous Cas9 function, making it suitable for genome engineering [26].

Therefore, this research aims to rationally identify DNA targets for PvdQ overproduction in *P. aeruginosa* PAO1 using a systems biology-based computational framework and to design highly efficient gRNAs for gene knockout via CRISPR-Cas9. The study also considers the impact on pyoverdine production with the main aim of engineering a strain that enhances QQ activity without unintentionally disrupting the balance of virulence factors. This study establishes an in silico framework grounded in previously validated models and bioinformatic tools, providing a rational basis for future experimental validation. This work contributes to the field of QQ-based antivirulence strategies while addressing critical biosafety considerations.

## Materials and Methods

### Rational target identification for gene knockout for PvdQ maximization

The selection of genes for knockout was conducted based on our previous work with the GEM iJD1249, composed of 1,249 genes, 1,051 proteins, 1,208 biochemical reactions, 205 exchange biochemical reactions (EBRs), 1,178 metabolites, and 3 compartments: cytoplasm, periplasm, and extracellular space (Tables S1 and S2) [21]. Two simulations were conducted applying flux balance analysis (FBA): simulation 1: biomass maximization (biochemical reaction 251), and simulation 2: biomass-PvdQ maximization (biochemical reactions 251 and 825). The in-house algorithm [27] developed by our research group was implemented in MATLAB (R2024b) (The MathWorks, Inc., Natick, MA, USA) to optimize specific metabolites. To perform the metabolic activity, the Luria–Bertani (LB) medium was selected for the in silico experiment because it is one of the most used media for genetic engineering purposes in *P. aeruginosa* PAO1 (Table S3). In addition to LB composition, QS molecules (3O-C_12_-HSL, C_4_-HSL, and PQS) were included to simulate QS activation (Table S4).

The selected concentration for the main QS signals, 3O-C_12_-HSL and C_4_-HSL, was 2.5 × 10^−3^ mM, as this amount has been identified as required for their activation in laboratory liquid media (BNID 112009) [28]. For PQS, 2 × 10^−3^ mM was selected as the representative concentration based on the known QS hierarchy, where PQS is present at lower levels than AHL systems. PQS concentrations range from 1 to 60 μM in liquid cultures, whereas sputum samples show markedly lower concentrations (0.3 to 36 nM) [29–31]. These values were selected to reflect controlled culture laboratory conditions, with the intention of enabling direct comparison in future in vitro validation studies.

Biochemical reactions from simulations 1 and 2 were filtered only if the change in carbon flux exceeded 90 units and the flux value was zero in simulation 2. This criterion was applied to reduce the large flux dataset (2,024 carbon fluxes per simulation) while prioritizing reactions exhibiting drastic changes between conditions. Specifically, reactions were required to transition from high carbon flux under biomass maximization (simulation 1) to complete inactivation (flux = 0) when biomass and PvdQ-associated reactions were jointly optimized (simulation 2). In this study, “PvdQ overproduction” refers to an increased flux through the PvdQ-catalyzed reaction and does not imply transcriptional up-regulation of *pvdQ*. A zero flux was interpreted as inactivation, indicating a redirection of metabolic flux toward PvdQ production while sustaining growth [32,33]. Importantly, the use of a 90-unit flux reduction threshold is supported by prior FBA-based analyses of metabolic perturbations in *P. aeruginosa*, in which it was effective in identifying biologically relevant changes [34].

The resulting reaction set was subsequently analyzed based on the GPR associations to identify the implicated genes. Only genes classified as nonessential for *P. aeruginosa* PAO1 were retained, based on the gene essentiality for this bacterium [35]. The final selection prioritized genes involved in QS-related pathways, including L-homoserine and L-methionine biosynthesis, *S*-adenosyl-L-methionine biosynthesis, palmitate biosynthesis II, and 2-heptyl-3-hydroxy-4(1*H*)-quinolone biosynthesis [21].

### Design of gRNA hits

The reference sequences of genes that allow PvdQ maximization and *pvdH* (PA2413, NC_002516.2) were retrieved from the NCBI [36] (https://www.ncbi.nlm.nih.gov/datasets/gene/; accessed on 2025 May 5). These sequences were used as individual input data in CHOPCHOP (https://chopchop.cbu.uib.no; accessed on 2025 May 6) using Cas9 nuclease parameters to design gRNA hits targeting a 20-nt complementary sequence to the 5′-end, positioned immediately upstream of the Protospacer Adjacent Motif (PAM) sequence (5′-NGG), for knockout in *P. aeruginosa* PAO1 [37,38]. The default parameters were used for all options, except for GC content, which was set between 60% and 70% to align with the GC content of the *P. aeruginosa* PAO1 (66.5%) and increase the gRNAs' efficiency [36,39].

### Rational identification of the most effective gRNA hits

The identification of the most effective gRNA hits from the bulk results, beyond the scoring and ranking of CHOPCHOP that considers self-complementarity, number of mismatches, and off-target sites, was guided by the nucleotide position efficiency parameters described by Radha [40] and applied to *P. aeruginosa* PAO1, where favorable nucleotide patterns are purines (A or G) that are strongly preferred within the last 4 nt of the gRNA, as they enhance gRNA-Cas9 binding. Cytosine (C) is preferred at position 16, and a high GC content at positions 4 to 8 enhances gRNA activity. In the canonical 5′-NGG PAM recognized by *Streptococcus pyogenes* Cas9, the “N” position is favored by a C. On the other hand, inefficient nucleotide positions include C at positions 3 and 20, G at position 16, and T at the position of the “N” in the PAM sequence that reduces targeting efficiency. The biological relevance of these parameters has been established from nonmicrobial systems, reflecting both the available experimental evidence and its current limitations [41–44].

A semiquantitative scoring system was developed to evaluate the efficient and inefficient features of gRNA hits. The score considers the biological relevance of each feature, with a weight assigned accordingly. For efficient features, one point was assigned for each favorable nucleotide at a given position (“Yes” = 1 point; “No” = 0 points). Features associated with gRNA activity or Cas9 binding received 2 points if present, while a favorable PAM, critical for Cas9 recognition, received 4 points due to its high impact. The maximum possible score for an optimal gRNA hit was 23. This score can be decreased with inefficient features: minus 4 points for unfavorable PAM and minus 2 points for each nucleotide known to reduce efficiency.

### Verification of gRNA to target DNA

The most effective gRNA hits were verified to analyze and visualize the structure and domains of the corresponding sgRNA sequences. This was accomplished using the CRISPR analysis web tool Synthego v1.3 (https://design.synthego.com/#/validate; accessed on 2025 June 16), which has been established in the literature as a reliable CRISPR tool resource for genome editing applications [45].

### Analysis of gRNA and sgRNA secondary structures

In addition to the rational identification of the most effective gRNA hits, analysis of their 2D structure is essential to shed light on their potential cleavage efficiency. sgRNA design was performed by appending the wild-type (WT) Cas9 gRNA scaffold sequence and the transcription termination residue, which is shown in bold, to the resultant top gRNA hits:

GTTTTAGAGCTAGAAATAGCAAGTTAAAATAAGGCTAGTCCGTTATCAACTTGAAAAAGTGGCACCGAGTCGGTGC**TTTT**-3′ [25].

The WT Cas9 gRNA scaffold was selected because it is in the majority of CRISPR plasmids in the Addgene repository, ensuring broad availability and compatibility with established gRNA design rules [46].

The RNA secondary structure analysis was conducted following the protocol by Hassan et al. [47], where gRNA 2D structures with Δ*G* values between −0.4780 and 0 kcal·mol^−1^ were considered as optimal due to their association with high cleavage efficiency [48]. The Δ*G* self-folding calculations and 2D structure predictions for gRNA and sgRNA were generated using the RNAfold server, a core program of the ViennaRNA package [49].

Positive controls were obtained from the Research Collaboratory for Structural Bioinformatics Protein Data Bank [50] (https://www.rcsb.org/; PDB IDs: 4OO8 and 8G1I; accessed on 2025 June 20) and were used specifically to compare the 2D structures and Δ*G* values of gRNAs and sgRNAs known to bind Cas9 from *S. pyogenes* [51]. The following notation was established: positive control 1 (PC_1_) corresponds to PDB ID 4OO8, and positive control 2 (PC_2_) corresponds to PDB ID 8G1I.

## Results

### Rational target identification for gene knockout for PvdQ maximization

The EBRs that exhibited variations included biomass, 2-oxoglutarate, CO_2_, palmitic acid, pyruvate, 3-hydroxybutyric acid, PQS, 3-oxo-dodecanoate, cis,cis-muconate, and ethanol (Table S5). Among those, palmitic acid (an end product of palmitate II biosynthesis), 3-oxo-dodecanoate (released by PvdQ as part of its QQ activity), and PQS were found to be the most important metabolites in identifying the carbon flux distribution and metabolite production when PvdQ was maximized. The corresponding EBRs are presented in Table 1.

**Table 1.** Exchange biochemical reactions. Simulation 1: biomass maximization; simulation 2: biomass-PvdQ maximization.

Exchange reaction	Simulation 1	Simulation 2
	[mmol (g DW h)^−1^]	
251, Biomass	0.4259 ^a^	0.3703 ^a^
1287, Palmitic acid	0.3855	0.0000
1324, PQS	0.0020	0.0000
1326, 3-oxo-dodecanoate	0.0025	18.2025

DW, dry weight

^a^ h^−1^.

The biomass of *P. aeruginosa* PAO1 remains positive, allowing its maximization alongside the PvdQ overproduction. Meanwhile, the exchange of palmitic acid and PQS is not excreted. The most notable result was the increase in 3-oxo-dodecanoate, the metabolite resulting from the PvdQ activity, which reached 18.2025 mmol (g DW h)^−1^. This suggests that 3-oxo-dodecanoate is excreted, but not homoserine lactone, the other hydrolysis product of 3O-C_12_-HSL.

The GPR association is shown in Table 2, where the identified genes are classified according to the GEM iJD1249. PA3182, PA4204, PA5439, PA3183, and PA4732 are associated with central metabolism; PA4519 is involved in amino acid biosynthesis; PA1421 is involved in amino acid degradation; and PA3633 is involved in cofactors, prosthetic groups, and electron carriers biosynthesis. None of the genes was identified as essential for *P. aeruginosa* PAO1; however, only the gene PA1806 (*fabI*) is involved in palmitate biosynthesis II, also known as the fatty acid biosynthesis II pathway (a QS-related pathway). Consequently, *fabI* (PA1806, NP_250497.1) was selected as the final knockout candidate, exhibiting strong potential.

**Table 2.** Gene–protein–reaction (GPR) associations for potential gene knockout. Simulation 1: biomass maximization; simulation 2: biomass-PvdQ maximization.

Biochemical reaction	Genes	Proteins	Simulation 1	Simulation 2
			[mmol (g DW h)^−1^]	
369	PA3182, PA4204	Pgl, PpgL	100	0
394	PA5439, PA3183	Zwf_p, Zwf	100	0
804	PA3633	YgbP	93.08	0
861	PA1470	–	91.06	0
1224	PA4519	SpeC	100	0
1298	PA1421	GbuA	96.38	0
1407	PA4732	Pgi	100	0
1450	PA3633	YgbP	93.08	0
1529	PA1806	FabI	90.68	0

### gRNA hits for *pvdH* and *fabI* knockout in *P. aeruginosa* PAO1

The gRNA hits for *pvdH* and *fabI* output a list of 146 and 78 target sequences, respectively (Figs. S1 and S2), which are reported in Tables S6 and S7 with their GC content (%), self-complementarity, number of mismatches, and predicted efficiency. The most efficient gRNA hits, filtered to exclude those with self-complementary sequences or mismatches, are summarized in Table 3. According to CHOPCHOP output, the top-ranked gRNA hit for *pvdH* has an efficiency score of 74.83; it is located at 2,694,954 in the reverse strand (−, negative), and has a GC content of 65%. For *fabI*, the most efficient gRNA hit has a score of 69.25; it is located at 1,961,346 in the reverse strand, and has a GC content of 70%.

**Table 3.** Target sequences for guide RNA (gRNA) for knockout of *pvdH* and *fabI* genes in *P. aeruginosa* PAO1

Gene	Rank by CHOPCHOP	Target sequence + **PAM**	Genomic location (NC_002516.2)	Strand	GC content (%)	Efficiency
*pvdH*	1	GGCCTGATCCTCGAACTGGG**CGG**	2,694,954	−	65	74.83
	2	GAACTGGTCCTTCACCGGGG**TGG**	2,695,907	+	65	67.43
	8	GTCGAGATCGTCGACCCGCA**GGG**	2,695,053	−	65	63.16
	13	CTGGTCTTCGATGACGCTGG**TGG**	2,696,219	+	60	60.17
	16	GTCAGGTCGAGGGTGTGCAG**CGG**	2,695,929	+	65	59.43
	17	TGGCTGGACAAATGGCAGCC**CGG**	2,695,269	−	60	59.40
	18	GGCTACCACGGCATGAGCCA**GGG**	2,695,734	−	65	58.42
*fabI*	1	GCGGCGCAACGTCACCATCG**AGG**	1,961,346	−	70	69.25
	2	TGTCGTCGGCCACGTCACAG**GGG**	1,961,814	+	65	69.08
	3	GGTGATCAGTCGTCGTCCAG**CGG**	1,961,222	+	60	66.19
	4	GCTGTGCTTCCCCTGTGACG**TGG**	1,961,823	−	65	64.31
	6	CGCTGGCCAGGTCCGAACAG**AGG**	1,961,301	+	70	63.69
	7	CACTGGCGAACTCCTCCACC**CGG**	1,961,865	+	65	63.00
	9	GCCCTGGGCAAGCACTGGGA**CGG**	1,961,768	−	70	60.32
	11	GTCCCAGTGCTTGCCCAGGG**CGG**	1,961,770	+	70	59.94
	12	TCTCCTACCTGGGCGCCGAA**CGG**	1,961,563	−	65	59.12

### Rational identification of the most effective gRNA hits

The efficient and inefficient features of the gRNA hits for *pvdH* and *fabI* genes are shown in Table 4. For the *pvdH* gene, gRNA hits Nos. 1, 2, 8, and 17 contain a cytosine at position 16. Sequences 2, 16, and 17 have a higher GC content. gRNA hits Nos. 1, 2, and 16, which contain purine residues at the 3ʹ end, are more likely to bind Cas9. The gRNA hits Nos. 1, 16, and 17 possess the efficient PAM (CGG). Among the analyzed sequences, gRNA hit No. 13 contains more than one inefficient feature.

**Table 4.** Efficient and inefficient features of gRNA hits for *pvdH* and *fabI* knockout

Gene	No. of gRNA hits	Efficient features				Inefficient features		
		C at position 16	GC at positions 4–8	Purine nt in the last 4 positions	PAM (CGG)	PAM (TGG)	G at position 16	C at position 3 or 20
*pvdH*	1	Yes	2 (**C**T**G**AT)	3 (T**GGG**)	Yes	No	No	Yes (3)
	2	Yes	3 (**C**T**GG**T)	4 (**GGGG**)	No	Yes	No	No
	8	Yes	2 (**G**A**G**AT)	2 (C**G**C**A**)	No	No	No	Yes (3)
	13	No	2 (**G**T**C**TT)	2 (CT**GG**)	No	Yes	Yes	No
	16	No	3 (A**GG**T**C**)	3 (**G**C**AG**)	Yes	No	No	Yes (3)
	17	Yes	3 (**C**T**GG**A)	2 (**AG**CC)	Yes	No	No	Yes (20)
	18	No	2 (TA**CC**A)	2 (**G**CC**A**)	No	No	No	Yes (3)
*fabI*	1	Yes	4 (**GCGC**A)	2 (**A**TC**G**)	No	No	No	No
	2	Yes	4 (**CG**T**CG**)	3 (**A**C**AG**)	No	No	No	No
3	No	2 (**G**AT**C**A)	2 (CC**AG**)	Yes	No	No	No
4	No	3 (**G**T**GC**T)	3 (**GA**C**G**)	No	Yes	No	No
6	No	4 (T**GGCC**)	3 (**A**C**AG**)	No	No	No	Yes (3)
7	Yes	4 (T**GGCG**)	1 (C**A**CC)	Yes	No	No	Yes (3 and 20)
9	No	4 (**C**T**GGG**)	4 (**GGGA**)	Yes	No	No	Yes (3)
11	Yes	3 (**CC**A**G**T)	4 (**AGGG**)	Yes	No	No	Yes (3)
12	Yes	3 (**CC**TA**C**)	3 (C**GAA**)	Yes	No	No	No

For the *fabI* gene, gRNA hits Nos. 1, 2, 7, 11, and 12 contain a cytosine at position 16. Sequences 1, 2, 6, 7, and 9 reveal a higher GC content. Purine residues at the 3′ end of the gRNA hits Nos. 9 and 11 are more likely to bind Cas9. Furthermore, gRNA hits Nos. 3, 7, 9, 11, and 12 possess the PAM CGG. Notably, gRNA hits Nos. 1, 2, 3, and 12 did not present any inefficient characteristics.

The semiquantitative scoring system used to evaluate the most effective gRNA hits is presented in Table 5. For the *pvdH* gene, gRNA hits Nos. 16, 1, 17, and 2, identified using CHOPCHOP, emerged as the most suitable gRNA hits, and for the *fabI* gene, the most suitable gRNA hits were Nos. 9, 11, 12, 2, and 1.

**Table 5.** gRNA hit efficiency scoring method targeting *pvdH* and *fabI*

Gene	No. of gRNA hits	C at position 16	GC at positions 4–8	Purine nt in the last 4 positions	PAM (CGG)	PAM (TGG)	G at position 16	C at position 3 or 20	Total score
*pvdH*	16	0	6	6	4	0	0	−2	14
	1	1	4	6	4	0	0	−2	13
	17	1	6	4	4	0	0	−2	13
	2	1	6	8	0	−4	0	0	11
	8	1	4	4	0	0	0	−2	7
	18	0	4	4	0	0	0	−2	6
	13	0	4	4	0	−4	−2	0	2
*fabI*	9	0	8	8	4	0	0	−2	18
	11	1	6	8	4	0	0	−2	17
	12	1	6	6	4	0	0	0	17
	2	1	8	6	0	0	0	0	15
	1	1	8	4	0	0	0	0	13
	3	0	4	4	4	0	0	0	12
	6	0	8	6	0	0	0	−2	12
	7	1	8	2	4	0	0	−4	11
	4	0	6	6	0	−4	0	0	8

### Verification of gRNA to gene target

The top 4 gRNA hits, Nos. 16, 1, 17, and 2, targeting the *pvdH* gene in *P. aeruginosa* PAO1, were selected for verification based on their total score. These 4 gRNA hits are effective sequences for targeting the *pvdH* gene. gRNA hits Nos. 16 and 2 are predicted to direct Cas9 and bind it to the antisense strand (−), while Nos. 1 and 17 bind the sense strand (+). The double-stranded DNA breaks (DSBs) are predicted to occur at the following genomic locations: gRNA 16 at 2,695,946 bp; gRNA 1 at 2,694,960 bp; gRNA 17 at 2,695,275 bp; and gRNA 2 at 2,695,924 bp (Fig. S3). They are predicted to have high activity (0.594, 0.748, 0.594, and 0.674, respectively) and show minimally predicted off-targets.

Similarly, the top 5 gRNA hits targeting the *fabI* gene in *P. aeruginosa* PAO1, Nos. 9, 11, 12, 2, and 1, were verified. These 5 gRNA hits were identified as effective sequences for targeting the *fabI* gene. gRNA hits Nos. 9, 12, and 2 are predicted to direct Cas9 and bind it to the sense strand, while gRNA hits Nos. 11 and 1 bind the antisense strand. The predicted DSBs are located at gRNA hit No. 9 at 1,961,774 bp; gRNA 11 at 1,961,787 bp; gRNA hit No. 12 at 1,961,569 bp; gRNA hit No. 2 at 1,961,352 bp; and gRNA No. 1 at 1,961,831 bp (Fig. S4). They are predicted to have high activity (0.603, 0.599, 0.591, 0.692, and 0.691, respectively) and show minimally predicted off-targets.

### Analysis of gRNA and sgRNA secondary structures

The gRNA 2D structures targeting *pvdH* (Nos. 16, 1, 17, and 2) and *fabI* (Nos. 9, 11, 12, 2, and 1) were analyzed. The minimum free energy (MFE) values of the gRNA hits are summarized in Table 6.

**Table 6.** gRNA hits and their minimum free energy values. PC1 and PC2 denote positive control 1 and 2, respectively.

Gene	No.	gRNA hits	MFE (kcal·mol^−1^)
*pvdH*	16	GTCAGGTCGAGGGTGTGCAG	0.00
	1	GGCCTGATCCTCGAACTGGG	−0.90
	17	TGGCTGGACAAATGGCAGCC	−6.70
	2	GAACTGGTCCTTCACCGGGG	−5.00
*fabI*	9	GCCCTGGGCAAGCACTGGGA	−5.20
	11	GTCCCAGTGCTTGCCCAGGG	−1.60
	12	TCTCCTACCTGGGCGCCGAA	−1.40
	2	TGTCGTCGGCCACGTCACAG	−1.70
	1	GCGGCGCAACGTCACCATCG	−2.50
Control	PC_1_	GGAAATTAGGTGCGCTTGGC	−0.10
	PC_2_	TACCAGCAAAACACTCCGAT	0.00

**Fig. 1.**
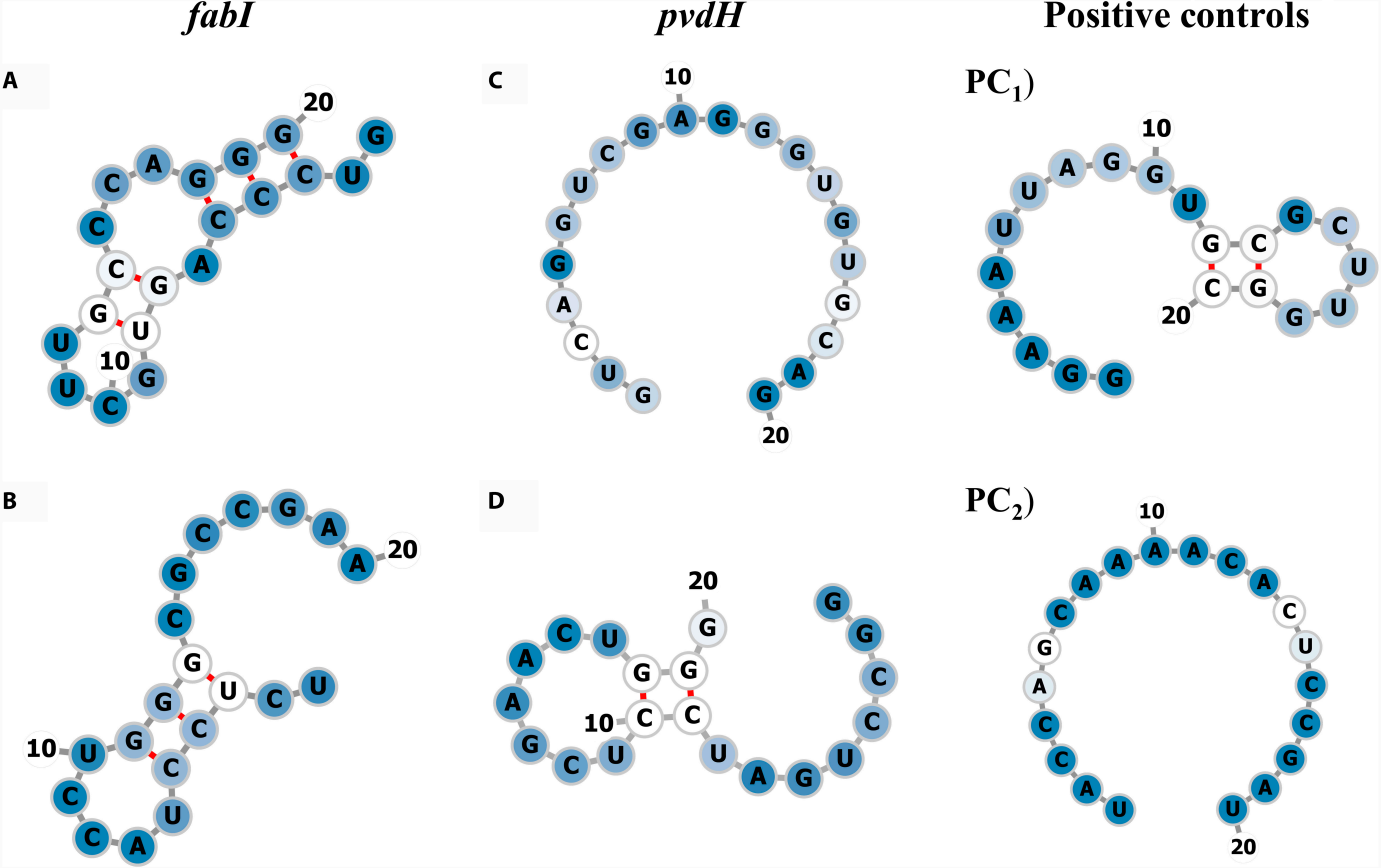
Predicted secondary structures of guide RNAs (gRNAs) targeting *fabI* and *pvdH*. For *fabI*, (A) gRNA No. 11 and (B) gRNA No. 12; for *pvdH*, (C) gRNA No. 16, (D) gRNA No. 1, (PC1) positive control 1, and (PC2) positive control 2. Base-pairing probabilities are represented by a white-to-blue gradient (0 to 1, where 1 indicates the highest probability). Red lines indicate base-paired regions within the self-folded structure. Numbers 10 and 20 are included for visual reference to nucleotide positions. RNA 2D structures were visualized with forna [72].

The predicted 2D structures of the top gRNAs with MFE values closest to the optimal range, targeting the *fabI* (No. 11 and No. 12) and *pvdH* (No. 16 and No. 1) genes, are shown in Fig. 1. Additional gRNA 2D structures are provided in Fig. S5, and the base-paired probability values for all gRNAs are summarized in Table S8. For *fabI*, gRNAs Nos. 12, 2, and 1 show an unpaired seed region; however, only gRNAs Nos. 12 and 1 have greater accessibility at the 3′-end of the gRNA, and notably, their predicted 2D structures are similar to that of PC_1_. Although gRNA No. 2 has an accessible seed region, its stable central structure may compromise the interaction with the target DNA. In contrast, gRNA No. 9 displays a seed region that is base-paired at positions 3 to 4 and is highly structured in the central region. gRNA No. 11 also presents an inaccessible seed region due to strong internal base pairing and a stable internal loop structure.

For *pvdH*, gRNA No. 16 has an unpaired seed region and displays a completely single-stranded structure. Notably, it is structurally similar to that of PC_2_. In comparison, gRNA No. 1 has paired nucleotides 18 to 19 with 10-9 (partially accessible), though with low base-pairing probability. Markedly, its predicted 2D structure is similar to that of PC_1_. The seed region in gRNA No. 17 is completely inaccessible for target DNA binding due to strong internal base pairing and self-folding (stable internal structure). Similarly, the 2D structure of gRNA No. 2 shows pairing between nucleotides at positions 18 and 4 (seed region partially inaccessible), which may negatively affect its functionality.

**Fig. 2.**
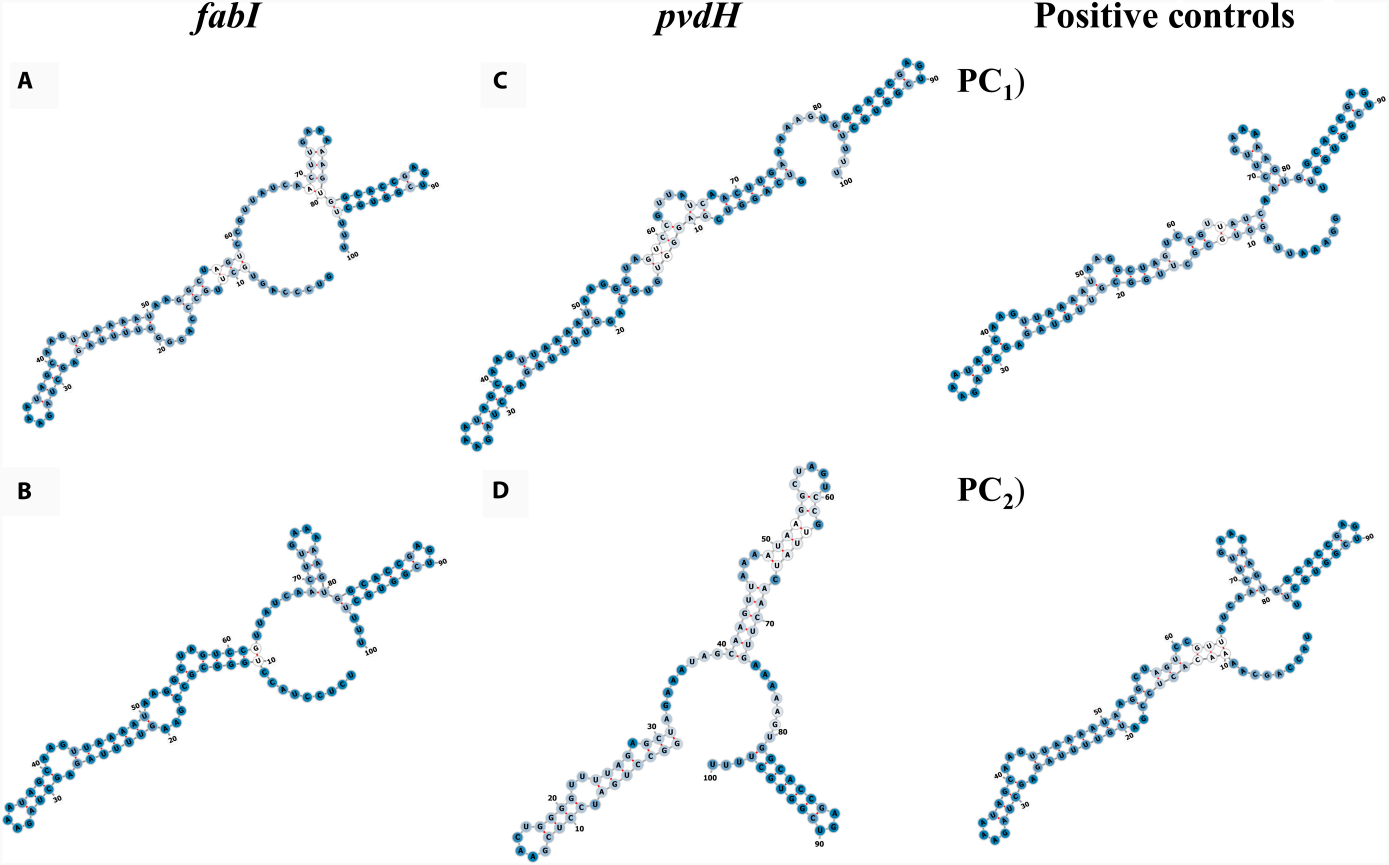
Predicted secondary structures of sgRNAs targeting *fabI* and *pvdH*. For *fabI*, (A) sgRNA No. 11 and (B) sgRNA No. 12; for *pvdH*, (C) sgRNA No. 16, (D) sgRNA No. 1, (PC_1_) positive control 1, and (PC_2_) positive control 2. Base-pairing probabilities are represented by a white-to-blue gradient (0 to 1, where 1 indicates the highest probability). Red lines indicate base-paired regions within the self-folded structure. Numbers are included for visual reference to nucleotide positions. RNA 2D structures were visualized with forna [72].

The structural features of the top sgRNAs targeting *fabI* (gRNAs Nos. 11 and 12) and *pvdH* (gRNAs Nos. 16 and 1) genes are shown in Fig. 2. Additional sgRNA 2D structures are provided in Fig. S6, and the base-paired probability values for all sgRNAs are summarized in Table S9. For *fabI,* all 5 top sgRNAs show the RAR loop, stem loop 2 containing the GAAA motif (nt positions 73 to 76), and stem loop 3 with the AGU motif (nt positions 88 to 90) (Fig. 2). None of the top sgRNAs present stem loop 1; however, it does not appear to impact functionality, as shown by the 2D structure of positive controls 1 and 2. sgRNAs Nos. 1, 2, 11, and 12 show base pairing at nucleotide position 53, while sgRNA No. 9 presents pairing at positions 52 and 53.

For the *pvdH* gene, the repeat and anti-repeat region (RAR) loop (nt positions 33 to 36) and stem loop 3 (nt positions 81 to 97) were predicted in sgRNAs Nos. 16, 17, and 2, showing structural similarity to positive controls PC_1_ and PC_2_. sgRNA No. 1 was the only guide that did not present the RAR loop. Only sgRNAs Nos. 17 and 2 exhibited stem loop 2 (nt positions 69 to 80), also consistent with the 2D structures observed in PC_1_ and PC_2_. None of the sgRNAs showed the presence of stem loop 1. Additionally, sgRNAs Nos. 16 and 17 presented the unpaired nucleotides at positions 51 to 53, forming an antiparallel configuration with the seed region (nt positions 18 to 20).

## Discussion

The *fabI* gene, identified as a rational target through FBA, encodes the FabI protein, an enoyl-acyl carrier protein [ACP] reductase that regulates acyl chain length. It is currently a focus of research aimed at inhibiting AHLs production, with the advantage of being nonhomologous to mammalian targets. The enzyme is involved in cell wall biosynthesis, particularly in lipids and fatty acid production, where palmitic acid is the most abundant higher fatty acid. FabI catalyzes the NADH-dependent reduction of enoyl-ACP intermediates to their corresponding acyl-ACP products [52,53].

**Fig. 3.**
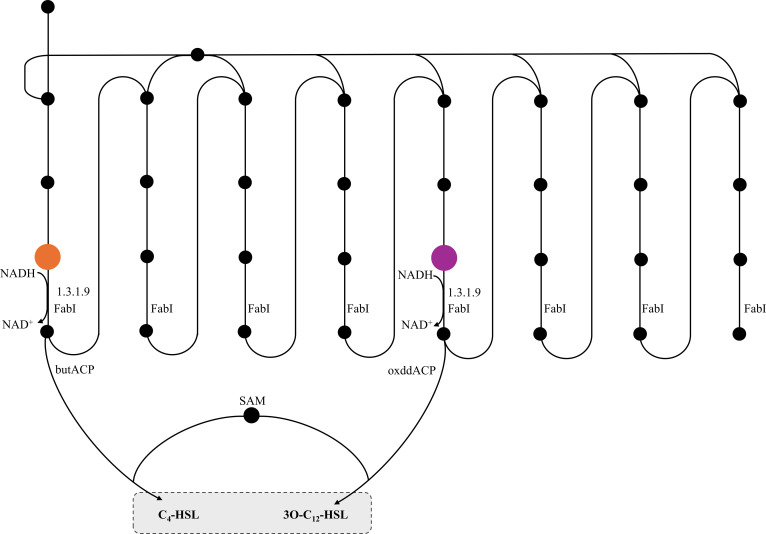
Involvement of FabI in the palmitate biosynthesis II pathway, also known as fatty acid biosynthesis II (FAS II) pathway, and its connection to AHLs production. Orange and purple circles represent but-2-enoyl-[ACP] and trans-dodec-2-enoyl-[ACP], respectively. Pathway information was obtained from *P. aeruginosa* BioCyc. Abbreviations: butACP, butyryl-ACP; oxddACP, 3-oxododecanoyl-ACP; SAM, S-adenosyl-L-methionine.

In the context of QS, FabI mediates the reduction of but-2-enoyl-[ACP] to butanoyl-[ACP] and facilitates the cycle necessary to produce the 3-oxo-dodecanoyl-[ACP] precursors required for C_4_-HSL and 3O-C_12_-HSL, respectively, thereby directly influencing the availability of fatty acid substrates required for AHL biosynthesis (Fig. 3). Consequently, disruption of FabI limits the pool of acyl-ACP intermediates, a mechanism that has been experimentally validated as an effective strategy to inhibit AHL synthesis [54,55].

Within this framework, FabI emerges as a strong predicted target in the palmitate biosynthesis II pathway owing to its essential role in acyl-chain elongation. Beyond these direct biosynthetic effects, since fatty acids influence the transcriptional regulator LasR, perturbations in FabI activity may indirectly modulate the expression of *pvdQ*, a QS-regulated gene [56,57]. In *P. aeruginosa*, QS coordinates a hierarchy of regulatory factors, including *lasI*, *rhlR*, *mvfR*, and *pvdQ*. This positions FabI at a strategic metabolic crossroads where the availability of AHL precursors can influence *pvdQ* expression, which likely intersects with stress-responsive regulators such as LasR, PvdS, and PhoB, which respond under conditions such as phosphate depletion and iron limitation [58].

In the context of CRISPR-Cas9 genome editing, the 2D structure of gRNAs plays a crucial role in determining target accessibility and cleavage efficiency. Structural analysis of gRNA hits targeting both *pvdH* and *fabI* exhibits features strongly associated with enhanced Cas9 activity. For targeting *pvdH*, gRNA No. 16 is the only top candidate whose MFE suggests it is a strong candidate for efficient cleavage. Same for targeting *fabI*, gRNA hit No. 12 is the top candidate closest to the optimal MFE range [48]. Both gRNAs have an unpaired seed region, a structural feature associated with higher cleavage efficiency [47]. These findings collectively support gRNA No. 16 for *pvdH* and gRNA No. 12 for *fabI* as the most promising candidates for effective genome editing using CRISPR-Cas9.

In addition to the gRNA 2D structure, a functional sgRNA contains 5 structural modules: the lower stem, upper stem, bulge, nexus, and hairpin. The absence of the hairpin structure disrupts the ability of the sgRNA:Cas9 to induce the DSBs. Among these modules, the bulge and nexus are particularly important for sgRNA composition. Some sgRNA variants have a tetraloop replacing the bulge structure [59]. The requirements for an effective sgRNA are a 4-stem loop structure, which includes the RAR stem loop (GAAA), along with stem loop 1, stem loop 2, and stem loop 3. In addition to structural configuration, other features also contribute to sgRNA efficiency, including the accessibility of nucleotides at positions 51 to 53 [47].

Based on MFE values and structural features, sgRNA No. 16 targeting *pvdH* and sgRNA No. 12 targeting *fabI* remain the best candidates for CRISPR-Cas knockout (Table 7).

**Table 7.** 2D structural features and modules of selected gRNAs and sgRNAs

Gene	No.		gRNA		sgRNA			
		Sequence	Seed region	Central region	RAR loop	Stem loop 1	Stem loop 2	Stem loop 3
*pvdH*	16	GTCAGGTCGAGGGTGTGCAG	Accessible	Single-stranded	Present	Not present	Not present	Present
*fabI*	12	TCTCCTACCTGGGCGCCGAA	Accessible	Structured	Present	Not present	Present	Present

sgRNA No. 16 (*pvdH*) and sgRNA No. 12 (*fabI*) contain the RAR loop, which ensures the availability of the sgRNA for binding to the Cas9 protein [47]. sgRNA No. 16 has unpaired nucleotides at positions 51 to 53, which are correlated with high Cas9 efficiency and form an antiparallel configuration with positions 18 to 20, which is one of the conserved structural features in functional sgRNAs [60,61]. Although sgRNA No. 12 presents the nucleotide 53 paired, this feature is also present in positive control 2. This suggests that it does not necessarily inhibit functionality. While sgRNA No. 16 has stem loop 3, it lacks stem loops 1 and 2. sgRNA No. 12 has stem loops 2 and 3, but it lacks stem loop 1. However, stem loop 1 is not directly associated with genome editing efficiency, whereas stem loops 2 and 3 contribute to stable sgRNA:Cas9 complex formation [62]. Moreover, stem loops 2 and 3 are known to tolerate a wide range of mutations and structural variations without compromising function [51].

The present study differs from previous work at both the modeling and implementation levels. GEM-based studies in *P. aeruginosa* PAO1 have largely focused on metabolic adaptation, nutrient utilization, or growth optimization; when QS was included, it was typically analyzed as a regulatory or metabolic outcome rather than as an engineering objective [63,64]. In contrast, we employed iJD1249 not to predict growth phenotypes, but to identify metabolic intervention points capable of redirecting flux toward endogenous PvdQ overproduction. This reframes the GEM from a descriptive tool to a platform for rational target discovery in antivirulence design.

Similarly, prior CRISPR-based strategies in *P. aeruginosa* have primarily targeted direct disruption of QS regulators or virulence-associated genes, such as *lasR*, *rhlR*, and *pelA* [40,65]. Here, rather than directly disabling QS, we propose a metabolic strategy that enhances endogenous QQEs. The integration of flux-based target prioritization with semiquantitative gRNA efficiency scoring and 2D structural evaluation further bridges computational prediction and experimental feasibility, establishing a coherent pipeline from GEM-driven analysis to CRISPR-Cas9 implementation [66].

## Conclusion

This study applied a systems biology approach to achieve a deeper understanding of *P. aeruginosa* PAO1 and to rationally identify DNA targets for QQE overproduction, with a particular emphasis on PvdQ. It was determined through GEM iJD1249 that a total of 10 genes that are knockout targets have the potential to affect the PvdQ overproduction. Among those, the *fabI* gene, involved in the palmitate biosynthesis II pathway, emerged as the most promising target. Its predicted knockout is expected to enhance PvdQ synthesis, thereby leading to biofilm reduction through increased QQ activity.

Based on biologically relevant criteria for Cas9 binding and target recognition, rational gRNA design combined with 2D structural validation identified gRNA hit No. 12 as the most effective candidate for *fabI* knockout and gRNA No. 16 for *pvdH* knockout. These findings define precise genome-editing targets for experimentally enhancing the intrinsic QQ capacity of *P. aeruginosa*.

The experimental validation of *fabI* and *pvdH* knockouts would provide a more profound understanding of new antivirulence strategies against MDR. Although beyond the scope of the present study, a streamlined framework for future in vitro validation may include confirmation of genome editing by PCR amplification using gene-specific primers. QQ activity may be evaluated through AHL degradation assay employing the *Agrobacterium tumefaciens* A136 biosensor strain for 3O-C_12_-HSL detection, together with PvdQ quantification by fast protein liquid chromatography [67,68]. Subsequent phenotypic characterization could encompass biofilm formation assays using crystal violet staining, structural analysis by confocal laser scanning microscopy to obtain 3-dimensional biofilm images, and scanning electron microscopy to assess changes in surface topology [69–71].

To the best of the authors’ knowledge, this is the first study to propose a QQ-based antivirulence strategy through endogenous PvdQ overproduction, guided by systems-level target identification and addressing critical biosafety evaluation. If *P. aeruginosa* PAO1 already encodes enzymes for QS disruption, such as PvdQ, leveraging these endogenous mechanisms could offer a novel therapeutic strategy, turning the pathogen’s systems against it, reducing selective pressure for resistance, and contributing to the fight against AMR.

## Data Availability

All relevant data are within the paper and the Supplementary Materials, and the code is available on a GitHub repository at https://github.com/delgado-nungaray/Flux-Balance-Analysis. We have also used Zenodo to assign a DOI to the repository: https://doi.org/10.5281/zenodo.18475563.
