# Supplementary material for: Rational Targeting and gRNA Design for Enhancing Quorum Quenching in Pseudomonas aeruginosa PAO1
Source: Comput Struct Biotechnol J. 2026 May 4;35(1):0089. doi: 10.34133/csbj.0089 (PMC13136621; doi:10.34133/csbj.0089)
Supplement: Supplementary 1 — Figs. S1 to S6 Tables S1 to S9 [file csbj.0089.f1.zip › Supplementary Material.docx]

**Supplementary Material**


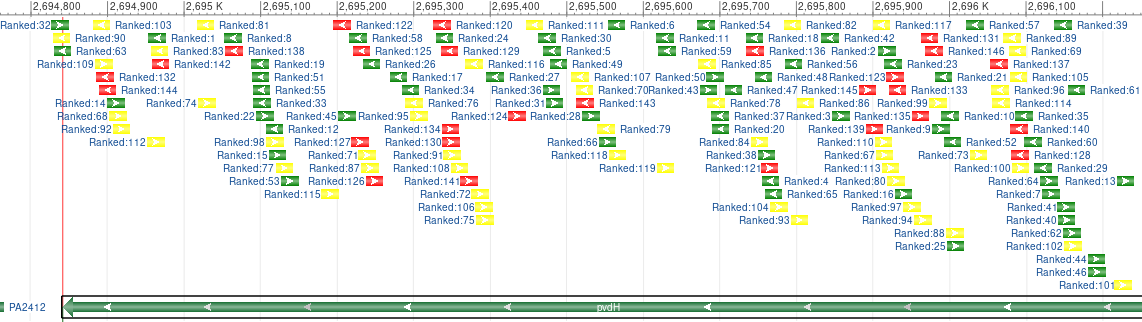


1. Genome view of the *pvdh* gene in *P. aeruginosa* PAO1 with identiﬁed gRNAs. The direction (right/left) is according to the DNA strand, according to the genomic sequence. Colour-coded arrows: green (best), yellow (regular), and red (bad). The figure was generated by downloading the BED annotation file from CHOPCHOP and uploading it as a new track into the NCBI Genome Data Viewer for the *P. aeruginosa* PAO1 genome.


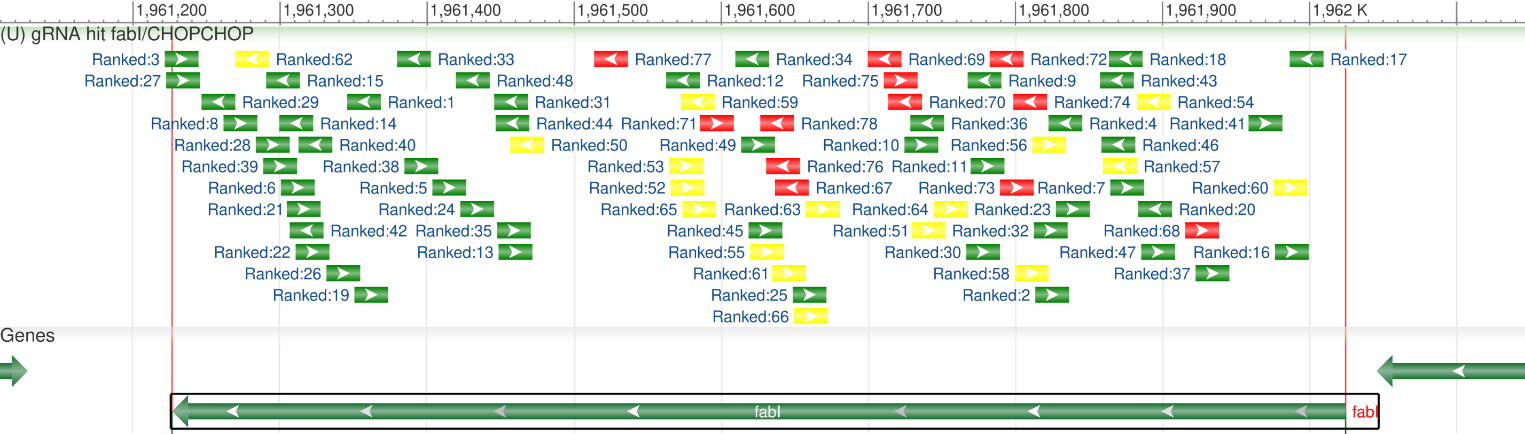


1. Genome view of the *fabI* gene in *P. aeruginosa* PAO1 with identiﬁed gRNAs. The direction (right/left) is according to the DNA strand, according to the genomic sequence. Colour-coded arrows: green (best), yellow (regular), and red (bad). The figure was generated by downloading the BED annotation file from CHOPCHOP and uploading it as a new track into the NCBI Genome Data Viewer for the *P. aeruginosa* PAO1 genome.


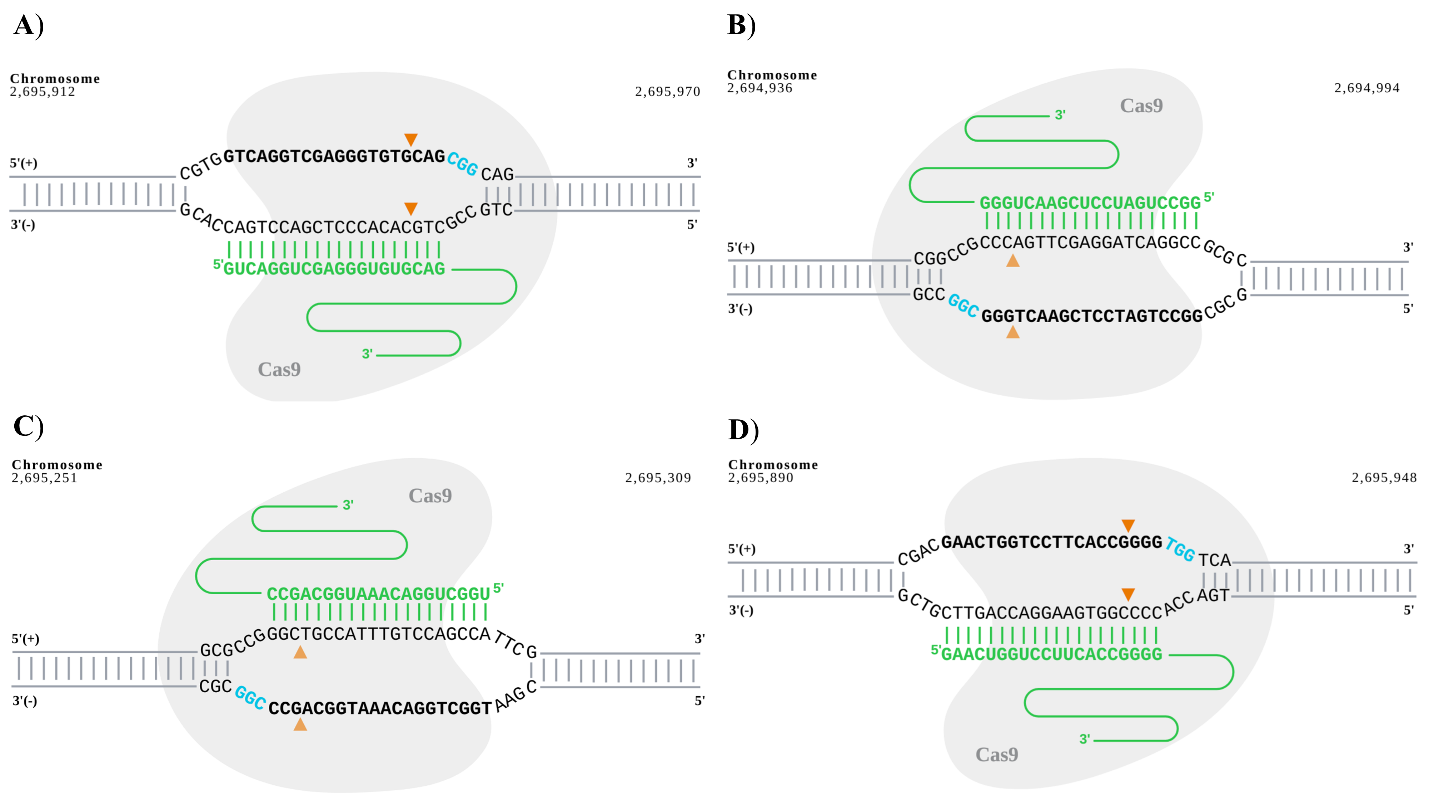


1. gRNA sequences targeting *pvdh* in *P. aeruginosa* PAO1. A) gRNA 16, B) gRNA 1, C) gRNA 17, and D) gRNA 2. The target site is highlighted in bold, the PAM region is shown in blue, the predicted Cas9 cut site is indicated by an orange triangle, and the sgRNA sequence is marked in green. Structures were visualised with Synthego v1.3.


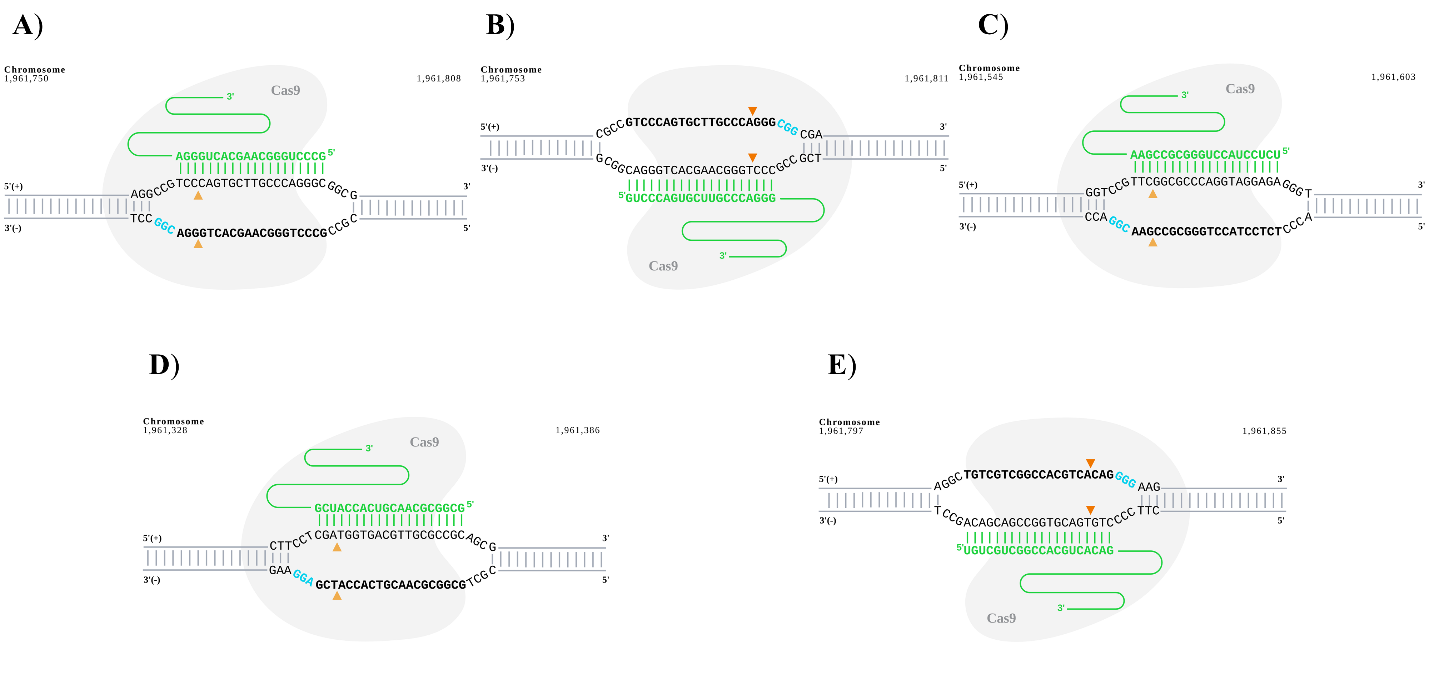


1. gRNA sequences targeting *fabI* in *P. aeruginosa* PAO1. A) gRNA 9, B) gRNA 11, C) gRNA 12, D) gRNA 2, and E) gRNA 1. The target site is highlighted in bold, the PAM region is shown in blue, the predicted Cas9 cut site is indicated by an orange triangle, and the sgRNA sequence is marked in green. Structures were visualised with Synthego v1.3.


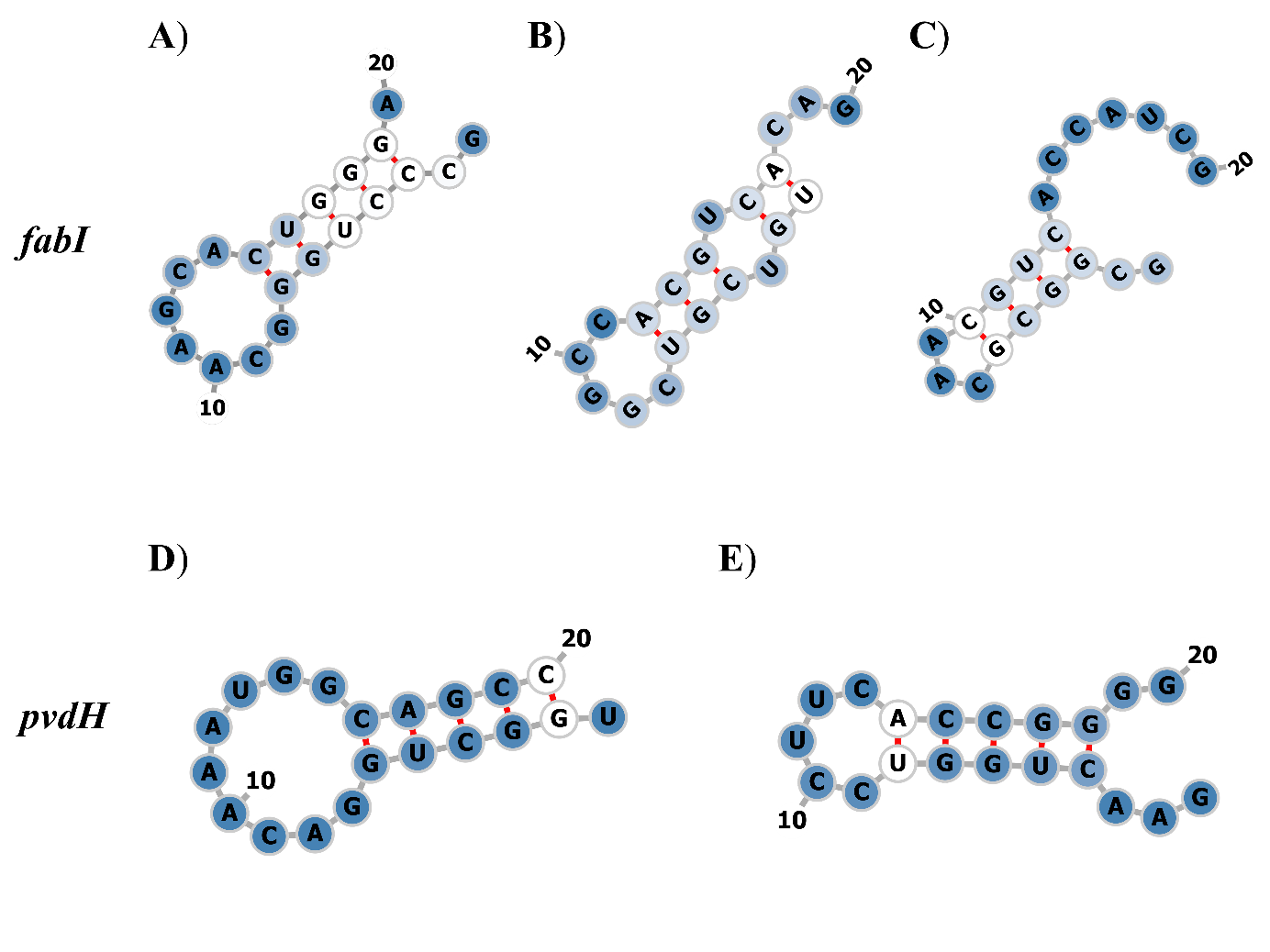


1. Predicted 2D structures of gRNAs targeting *fabI* and *pvdh*. For *fabI*, A) gRNA No. 9, B) gRNA No. 2, C) gRNA No. 1; for *pvdh*, D) gRNA No. 17, E) gRNA No. 2. Base-pairing probabilities are represented by a white-to-blue gradient (0-1, where 1 indicates the highest probability). Red lines indicate base-paired regions within the self-folded structure. Numbers 10 and 20 are included for visual reference to nucleotide positions. RNA 2D structures were visualised with forna.


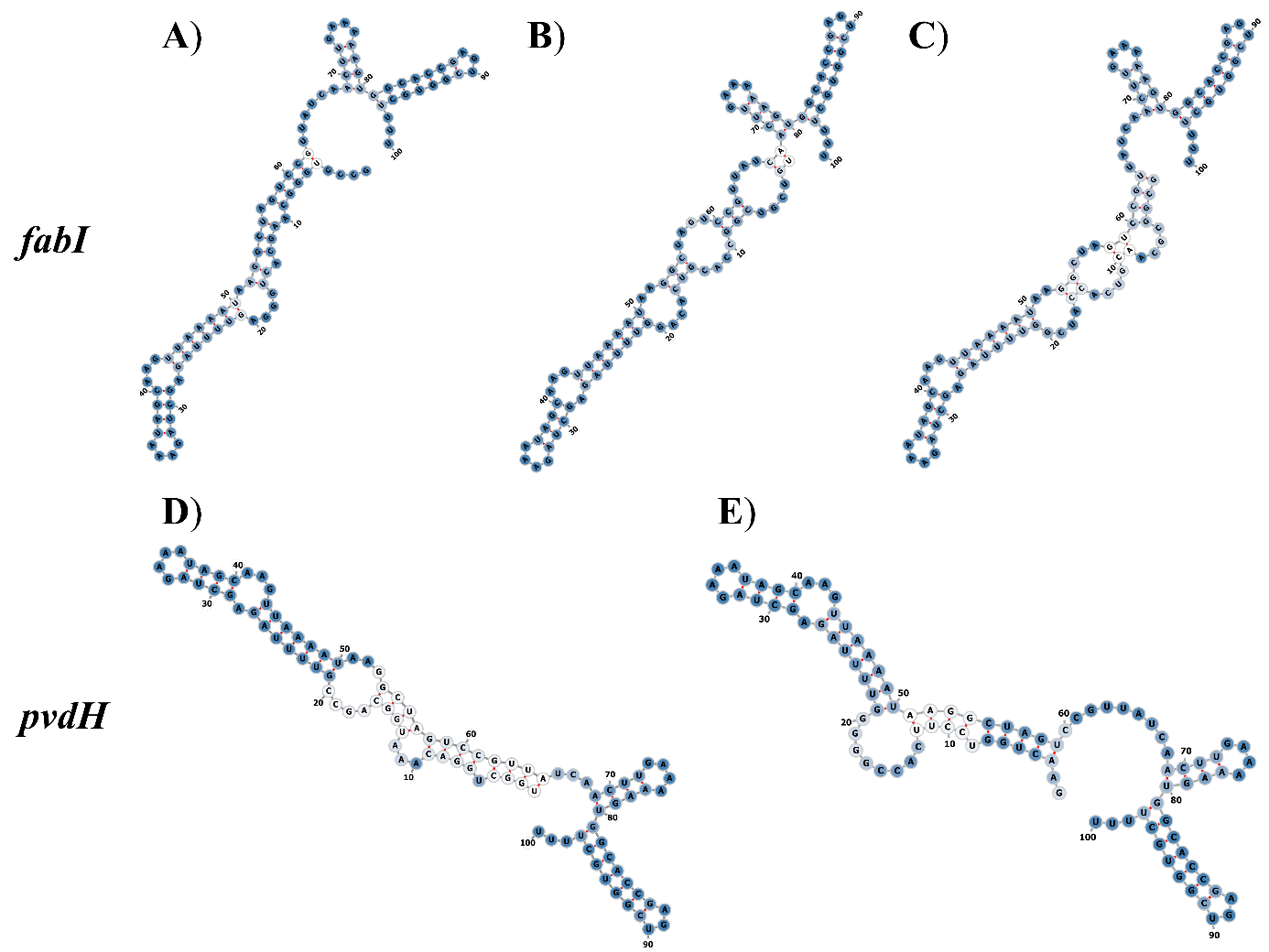


1. Predicted 2D structures of sgRNAs targeting *fabI* and *pvdh*. For *fabI*, A) gRNA No. 9, B) gRNA No. 2, C) gRNA No. 1; for *pvdh*, D) gRNA No. 17, E) gRNA No. 2. Base-pairing probabilities are represented by a white-to-blue gradient (0-1, where 1 indicates the highest probability). Red lines indicate base-paired regions within the self-folded structure. Numbers are included for visual reference to nucleotide positions. RNA 2D structures were visualised with forna.
